# Feel connected to create: Self-reported psychedelic drug users exhibit higher sense of connectedness and better divergent thinking skills compared to non-users

**DOI:** 10.1371/journal.pone.0320755

**Published:** 2025-04-11

**Authors:** Christian Rominger, Carolina Subow

**Affiliations:** Department of Psychology, University of Graz, Graz, Austria; University of Brescia: Universita degli Studi di Brescia, ITALY

## Abstract

Psychedelic drugs can increase health, wellbeing, and even boost cognitive functions such as creativity. Beyond this, previous studies indicated that psychedelic drug intake can increase the sense of connectedness to the world, to others, and to the self. The present preregistered cross-sectional online survey investigated whether the link between psychedelic drug use and creativity (as a potential and real-life creativity) takes place due to the increased sense of connectedness in psychedelic drug users. We collected data of 326 participants (187 psychedelic users and 139 non-users), who worked on an alternate uses task and answered questionnaires assessing real-life creativity, sense of connectedness, the experience of meaningful coincidences, as well as life satisfaction and affect. In line with all preregistered hypotheses, we found that psychedelic drug users showed a higher sense of connectedness, higher creative potential (i.e., originality, fluency), and more creative activities (at a trend). Furthermore, feelings of connectedness (to the self and to the world) were associated with the originality of ideas and real-life creativity, and connectedness to the self partially mediated the difference in the originality of ideas between the psychedelic drug users and non-users. Life satisfaction and positive affect were not significantly higher in psychedelic users but were positively linked to connectedness, creativity, and to synchronicity experiences. These findings provide evidence for the association between self-reported psychedelic drug use and creativity and strengthen the role of connectedness (to the self) as a potential psychological reason why psychedelics might enhance creativity.

## Introduction

There is a renaissance of studies of the psychological effects of drugs such as psilocybin, mushrooms, and LSD with a continuously improving methodological quality and rigor [1]. Psychedelic drug intake seems to benefit health and mood with the potential to decrease the symptoms of depression and anxiety [2]. These effects may also impact cognitive functions [3–5]. Studies investigating the acute effects of psychedelics on creative cognition, defined as the ability to generate original and useful ideas [6], showed negative effects at full doses. The longer-term effects, however, appear to be more positive for creative cognition ([5,7–10]; for a null effect see [11]; for reviews see, e.g., [1,12]). Furthermore, studies also indicated positive effects for lower than full doses [3] as well as for microdosing, which is taking a dose at sub-perceptual levels, on creative ideation performance ([13–15]; for critical reviews and meta-analyses see [12,16,17]). But why, if at all, are psychedelic drug use and creative cognition associated, and does the finding of increased creative ideation performance generalize to more real-life creative behavior such as creative activities and achievements?

Besides biological factors (e.g., serotonin agonist; [1]), the effects of enhanced mood [18], and pattern break [3], might an increased sense of connectedness [19,20] be a further potential psychological explanation for the association between psychedelic use and creativity? The feeling of connectedness – to the self, to others, and to the world - increases after psychedelic drug intake and people who use psychedelic drugs (e.g., psilocybin) might show higher scores on connectedness in general [19,21]. In line with this, studies indicated that more creative people tend to have larger social networks and stronger friendships [22,23]. People are more creative when more connected to others, and the connection to others seems important to reach goals and to develop creativity [24]. This conclusion is in accordance with Mahon et al. [25], who showed a negative link between loneliness and creativity across three independent samples. Similarly, Tan et al. [26] reported that the induction of social support can enhance creativity. Furthermore, Kaufman [27] suggested that both creativity and connectedness are relevant for finding meaning in life and achieving life satisfaction [28].

Furthermore, studies explored the relationship between natural environments and creativity [29–31]. For instance, Leong et al. [32] found a positive correlation between the feeling of connectedness with nature and more innovative and holistic cognitive styles. In their preprint, the authors also highlighted a connection between higher connectedness with nature and creativity [33]. They argued that the ability to adopt the perspectives of others, as suggested by the perspectival model [34], may facilitate the generation of novel and useful ideas. Connectedness to the self and to the own emotions might signal similar skills to take different perspectives. In line with this, better interoceptive skills, emotion regulation, and self-regulation skills in people more connected to themselves might lead to higher creative ideation performance, engagement in creative activities, and more creative achievements [35,36].

Connecting distant elements with each other to create novel combinations, which are original and useful is important for creativity [37,38]. Therefore, the experiences of meaningful coincidences (synchronicity experience) is an additional psychological concept potentially associated with psychedelic drug use, the sense of connectedness, mystical experiences (characterized by feelings of timeless and an absolute sense of unity), as well as creativity [2,19,39,40]. Meaningful coincidences are the experience of a tight connection between events, which are not causally related - such as the moment when a dream we had last night comes true [41,42]. The moment we recognize this connection is often accompanied by the epistemic emotion of surprise [43]. The perception of meaning in random events is associated with relevant traits such as a higher frequency of everyday creative activities and more creative achievements [39], as well as higher life satisfaction [44], and more positive affect [45]. Therefore, we conducted an exploratory analysis to determine if people who experience more meaningful coincidences are more likely psychedelic drug users, show a higher sense of connectedness, and have a higher potential to arrive at more creative ideas because over inclusiveness and hyper associative states might enhance creativity [46,47].

As preregistered [48], we hypothesized that (H1) people with self-determined psychedelic experiences (i.e., psychedelic drug users) would show higher connectedness scores compared to non-users [19]. We furthermore hypothesized that (H2a) users would outperform non-users in creativity tasks (i.e., alternate uses [AU] task; e.g., [13,15]). In exploratory analyses, we investigated if the findings generalize to real-life creative activities and achievements. We further explored if the sense of connectedness (with the self, others, and the world) is associated with creativity [33], and whether it could mediate the association between psychedelic use and creative ideation performance. This investigation aimed to identify the sense of connectedness as a possible psychological explanation for the suggested link between psychedelic drug use and enhanced creative cognition. As part of our hypotheses, (H2b) we also assumed a positive association between the frequency of psychedelic drug use and creative cognition (i.e., originality, and fluency). Additionally, we conducted exploratory analyses to investigate associations of these variables with well-being (i.e., positive and negative affect) and life satisfaction. Although not the main aim of this study report we have also preregistered (H3) that a stronger sense of connectedness is associated with diminished addictive behavior.

## Materials and methods

### Participants

A total sample of 326 participants (179 women, 136 men, 11 diverse) worked on the AU-task (772 participants followed the survey link and 42.23% provided sufficient information during the survey, i.e., AU-task performance measure). In sum, 187 participants were psychedelic users (57.4%), and 139 participants were non-users (42.6%) according to their self-reports. All participants were older than 18 years and the mean age of the sample was 29.58 years (*SD* =  9.74; for more details on the group characteristics see Table 1). All participants gave informed consent before participating in the online survey. The ethics committee of the university of Graz approved the preregister study [48] (GZ. 39/103/63 ex 2022/23). For some analyses such as addiction (H3; n = 303), life satisfaction, and well-being (exploratory analyses; n = 309) the sample size was lower.

**Table 1:** Demographic variables.

	Users		Non-Users		t/Chi (p)	
	**187**	57%	**139**	43%		
**Gender**					<.001	
Women	**74**	39%	**105**	76%		
Men	**104**	56%	**32**	23%		
Diverse	**9**	5%	**2**	1%		
**Age**	**30.9 (8.91)**		**27.8 (10.5)**		.004	
**Education**						
No qualification	**0**	0%	**1**	0%	.012	
Compulsory/Basic secondary school	**12**	7%	**4**	3%		
Intermediate secondary school certificate	**20**	11%	**4**	3%		
High school diploma/A-levels	**64**	34%	**72**	52%		
Bachelor’s degree	**47**	25%	**28**	20%		
Master’s degree	**26**	14%	**20**	14%		
Doctorate (PhD)	**6**	3%	**5**	4%		
Other	**12**	6%	**5**	4%		
**Employee**						
Student	**62**	33%	**76**	55%	<.001	
Full-time	**45**	24%	**34**	24%		
Part-time	**33**	17%	**11**	8%		
School student	**3**	2%	**3**	2%		
Unemployed/Job-seeking	**21**	11%	**3**	2%		
Retiree/Pensioner	**1**	1%	**2**	2%		
Other	**22**	12%	**10**	7%		
**Country**						
Germany	**65**	35%	**35**	25%	.195	
Austria	**119**	63%	**101**	73%		
Swiss	**1**	1%	**0**	0%		
Other	**2**	1%	**3**	2%		
**Environment**						
Rural	**36**	19%	**27**	19%	.701	
Small town	**63**	34%	**55**	40%		
Medium-sized town	**57**	30%	**37**	27%		
Large city	**31**	17%	**20**	14%		
**Psychiatric diagnosis**	**69**	37%	**35**	25%	.034	
**Drug consumption**	**LTU**	**L3M**	**LTU**	**L3M**	p (LTU)	p (L3M)
Alcohol	**186** (99%)	**152** (81%)	**129** (93%)	**110** (79%)	.003	.733
Tobacco	**183** (98%)	**140** (75%)	**104** (75%)	**55** (40%)	<.001	<.001
Cannabis	**187** (100%)	**136** (73%)	**91** (65%)	**33** (24%)	<.001	<.001
Stimulants	**164** (88%)	**65** (35%)	**30** (22%)	**9** (6%)	<.001	<.001
Cocaine	**139** (74%)	**48** (26%)	**18** (13%)	**5** (4%)	<.001	<.001
Opiates	**63** (34%)	**11** (6%)	**2** (1%)	**0** (0%)	<.001	.009
Benzodiazepines	**57** (30%)	**14** (7%)	**8** (6%)	**3** (2%)	<.001	.059
Psychedelics	**186** (100%)	**82** (44%)	**0** (0%)	**0** (0%)	<.001	<.001
Dissociatives	**97** (52%)	**40** (21%)	**6** (4%)	**3** (2%)	<.001	<.001
New psychoactive substances (NPS)	**63** (34%)	**10** (5%)	**2** (1%)	**0** (0%)	<.001	.015
Other	**45** (24%)	**12** (6%)	**9** (6%)	**1** (1%)	<.001	.021

LTU = Lifetime use; L3M = Last 3 months.

### Deviations from pre-registration

We need to outline some deviations from the pre-registered protocol. First, we arrived at a final sample of 326 in contrast to 350 preregistered participants. We recruited a high number of participants (~770), however, only 326 participants worked on the online AU task (at least for one item). Since the sample size only slightly deviates from the a priori power analysis it should not strongly affect study results. The sample of 326 participants is large enough to detect effects of a medium size (*d* = .31) with a power of.80 and an alpha of .05. Second, to analyze the data, we preregistered t-tests and correlations. However, due to characteristics of the two independent samples of users and non-users, which differ in demographics (see Table 1), we additionally calculated more complex regression analyses, to statistically control for gender, age, education, diagnosis, as well as employment.

To evaluate H3, which was not of main interest of this study, we calculated a t-test instead of the preregistered correlation. Due to the data structure of the addiction scale this statistical analysis seems more robust than the preregistered correlation approach.

### Online survey method

The online questionnaire was available through a link. The second author (C.S.) employed snowball recruiting, initially sharing the link within private circles and requested further distribution. Additionally, C.S. utilized the email distribution service of the University of Graz and posted on the Swiss drug forum *eve&rave*, which promotes responsible and self-responsible drug use, to invite participants in the online survey. C.S. continuously encouraged participation from October 2023 until the end of February 2024. The data collection ended in March 2024.

### Psychedelic substance use

Participants who indicated they had had at least one experience with classic psychedelics were categorized as *users* otherwise participants were categorized as *non-users*. Participants with psychedelic experiences were asked to select the estimated frequency of their experiences (Once, 2-5 times, 6-10 times, 11-20 times, 21-50 times, 51-100 times, and more than 100 times). Afterwards, users were asked to recall their last particularly relevant psychedelic experience and indicate which dose they used (Microdose, light trip, medium trip, strong trip; in relation to a full dose, which is equivalent to 100 micrograms of LSD or approximately 2 grams of psilocybin-containing mushrooms), whether they had an intention for their trip before consumption (yes, no), and in which setting they spent this trip primarily (mostly indoors, mostly outdoors in nature, mostly outdoors in an urban area, mixed, or festival setting). Finally, they rated their overall psychedelic experiences on a 5-point Likert scale (from very negative to very positive).

### Additional demographic variables

We assessed participants’ education, employment, country, environment (place of residence), and asked for psychiatric diagnosis as well as the frequency of drug consumptions (e.g., alcohol, cocaine, cannabis) within lifetime and within the last three months.

### Watts connectedness scale (WCS)

The WCS assesses the feeling of connectedness [19]. For this study, we translated the English version into German using Google Translate. We proofread the translations and corrected them if needed. The scale has three dimensions: (1) connectedness to the self (CTS), (2) connectedness to others (CTO), and (3) connectedness to the world (CTW). The sum of the subscales is the general feeling of connectedness (WCS). The WCS consists of 19 items (CTS and CTO have six and CTW has seven items) that are rated on a 10-point Likert scale from 1 (not at all) to 10 (completely). The WCS shows good internal consistency (Cronbach’s alpha of.91 for CTS; α=.74 for CTO, and α=.89 for CTW). The mean score was 6.75 (*SD* = 1.66) for the sum score (CTS: *M* = 7.28, *SD* = 1.77; CTO: *M* = 7.07, *SD* = 1.65; CTW: *M* = 5.89, *SD* = 2.40).

### Synchronicity awareness and meaning-detecting

To assess meaningful coincidences, we used the German translation of the Synchronicity Awareness and Meaning-Detecting (SAMD) scale [39,44]. We used the synchronicity awareness (SA) subscale, which is similar to the coincidence questionnaire [39,49], the SA subscale refers to awareness of the occurrence of synchronicity events and involves 7 items (6-point Likert-Scale from 0 [never] to 5 [all the time]). Cronbach’s alpha was .82. The SA scale is the main target variable of this study (*M* = 3.94, *SD* = 0.81). Meaning detection (MD) was assessed with 13 items on a 7-point Likert scale (Cronbach’s alpha = .88). This subscale is not relevant for the present study.

### Positive and negative affect

Participants answered 20 items of the German version of the Positive and Negative affect Schedule (PANAS; [50]) on a five-point Likert scale from 1 (not at all) to 5 (very much). Cronbach’s alpha of the positive affect (α=.89) and negative affect were good (α=.88). The mean rating of positive affect was *M* = 3.33 (*SD* = 0.73) and of the negative affect was *M* = 2.11 (*SD* = 0.72).

### The satisfaction with life scale (SWLS)

The SWLS [51] assesses general life satisfaction via five statements which have to be rated on a 7-point Likert scale ranging from 1 (strongly disagree) to 7 (strongly agree). The overall satisfaction score is the sum of answers with *M* = 4.89 (*SD* = 1.27; α=.85).

### Creative ideation performance

We used the alternate uses (AU) task as a common procedure for measuring divergent thinking skills [52]. The AU task requires producing as many original uses of an everyday object as possible. We used the objects *bucket* and *pillow.* Participants had three minutes for each item and had the task of producing as many and as creative ideas as possible. Four independent raters (two men and two women) evaluated the originality of ideas on a 4-point Likert scale ranging from 1 (not original) to 4 (very original) based on the likelihood of someone would produce this idea (i.e., originality). Raters were instructed in the rating procedure and had to take the usefulness into account. C.S. manually removed duplicates in the participants’ responses and merged similar ideas in order to reduce the burden of ratings [53]. The ratings of originality showed moderate to good interrater agreement, with .73 for the *bucket* item and .64 for the *pillow* item. We calculated mean originality scores and fluency scores per person. Originality was the mean of all originality scores of ideas per person (*M* =  1.74, *SD* =  0.26) and fluency was the mean number of generated (non-redundant) ideas per person (*M* =  7.29, *SD* =  3.61).

### ICAA: the inventory of creative activities and achievements

We assessed creative activities (CAct) and creative achievements (CAch) with the Inventory of Creative Activities and Achievements (ICAA; [54]). The questionnaire asks for 8 different domains of creative activities and achievements (i.e., literature, music, arts and crafts, cooking, sports, visual arts, performing arts, science and engineering). The creative activities and creative achievements sum scores showed good Cronbach’s alpha of .80 and .71, respectively.

### SSBA: the screener for substance and behavioral addictions

The SSBA [55] is a short screening instrument to identify self-attributed addiction problems in four substances (tobacco, alcohol, cannabis, and cocaine) and six behaviors (gambling, shopping, video games, overeating, sexual activity, and work). We added the category of *other substances*. For each class of addiction (e.g., cocaine) participants had to answer four statements, each describing a potentially problematic behavior. Think about the last 12 months, how often did the following statements apply to you: (1) *I did it too often*, (2) *Once I started, I couldn’t stop*, (3) *I felt like I had to do it to function*, and (4) *I continued to do it even though it caused problems*. Participants rated these statements on a 5-point Likert scale from 0 (never) to 4 (always), with the additional response option of *does not apply to me* and *do not know/do not want to answer*. We calculated a mean score for each addiction, which we evaluated using the thresholds from Schluter and colleagues to determine if self-attributed addiction was present in the different areas. The threshold for behavioral addictions (except shopping and video games) was two and for all substance addictions (and the remaining behavioral addictions) was three. Internal consistency was good (α > .73), except for gambling (α = .66). We categorized participants as addicted (1 vs. non-addicted 0) if they scored above the threshold in any substance or behavior. This procedure resulted in 121 participants categorized as addicted and 182 as non-addicted.

### Statistical analyses

First, we provided detailed descriptive statistics on the characteristics of the two groups (psychedelic users vs. non-users) as well as more details on the characteristics of the group of psychedelic users.

Second, we used a t-test for independent samples to assess the effects of psychedelics (users vs. non-users) on connectedness (H1). Furthermore, we calculated exploratory Spearman Rho correlations between the frequency of psychedelic drug use and connectedness. Additionally, we calculated sensitivity analyses by means of a linear regression to indicate if results are due to differences in group characteristics such as age, gender, education, employment.

Third, we tested the H2a with a t-test for independent samples and sensitivity analyses by means of linear regressions. For H2b, we calculated Spearman Rho rank correlations between creativity indices (i.e., originality, fluency) and the self-rated frequency of psychedelic substance use.

Fourth, we additionally calculated exploratory analyses such es differences between users and non-users with respect to creative activities and creative achievements (using t-tests) as well as Pearson correlations between connectedness and creativity measures. We calculated sensitivity analyses by means of a linear regression to indicate if results hold true when controlled for group characteristics such as age, gender, education, employment.

Fifth, we calculated an exploratory mediation analysis of connectedness on the effect of psychedelic use on creativity (for originality). We used nonparametric bootstrapping with 1000 iterations by means of the mediation package (Version 4.5.0; [56]).

Finally, we explored the associations between variables of interest (i.e., psychedelic use, creativity, connectedness) and well-being (i.e., positive and negative affect) as well as life satisfaction. We again calculated sensitivity analyses by means of a linear regression to indicate if results hold true when controlled for group characteristics such as age, gender, education, employment.

All analyses were calculated in R (Version 4.4.1; [57]). We kept the level of significance fixed at *p* < .05 (two-tailed).

## Results

### Descriptive analyses

As illustrated in Table 1 the two groups (psychedelic users vs. non-users) were different with respect to gender. Men were more likely users than non-users (104 vs. 32) and women were more likely non-users than users (105 vs 74). Additionally, psychedelic users were three years older on average than non-users. Users had higher degrees of education, were more likely to be without employment, and had more psychiatric diagnoses. According to their self-reports, psychedelic drug users consumed more drugs during the last three months as well as during their lifetime.

Table 2 illustrates the frequency of psychedelic drug use as well as doses, intentions, and settings of their most recent and personally relevant psychedelic experience, and the subjective ratings of the quality of psychedelic drug experiences in general. Most people (69%) used psychedelics between once and 11-20 times in their life and had mostly positive experiences (90%) with psychedelics. Most participants took a medium trip (51%), with an intention (65%). The indoor setting (36%) was the most frequently reported setting followed by nature (30%).

**Table 2:** Descriptive statistics of experiences of psychedelic drug users.

		N	%
	Once	9	5%
	2-5 times	44	23%
**Frequency of psychedelic drug experiences**	6-10 times	40	21%
	11-20 times	37	20%
	21-50 times	20	11%
	51-100 times	22	12%
	More than 100 times	15	8%
	Very negative	0	0%
	Mostly negative	1	1%
**Quality of experience**	Neutral	17	9%
	Mostly positive	82	44%
	Very positive	87	46%
**Dose** ^*^	Microdose (10%)	5	3%
	Light trip (25-75%)	41	22%
	Medium trip (75-150%)	96	51%
	Strong trip ( > 150%)	44	24%
**Intention**	No	65	35%
	Yes	122	65%
	Indoor	67	36%
	In nature	56	30%
**Setting**	In urban environment	8	4%
	Different settings	35	19%
	Festival	21	11%

The dose refers to the amount for a full trip (equivalent to 100µg of LSD or 2g of dried mushrooms).

* The answer of one participant is missing.

H1: users of psychedelic compounds have a higher sense of connectedness compared to non-users of psychedelics

**Fig 1:**
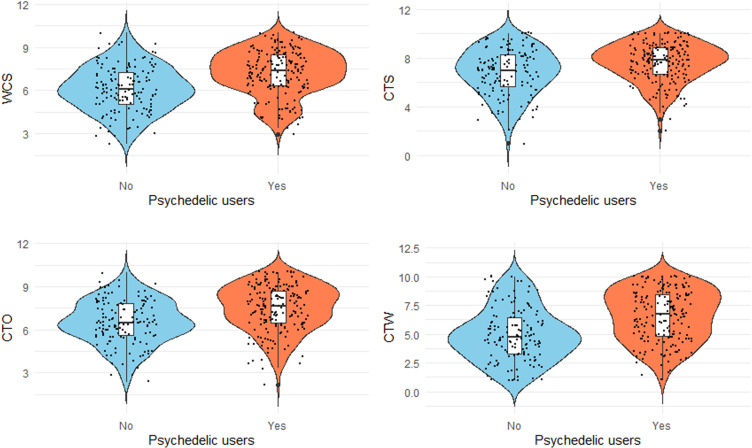
Violin plots for the sense of connectedness (WCS, top left) as well as the three sub scores, connectedness to the self (CTS, upper right), connectedness to others (CTO, bottom left), and connectedness to the world (CTW, bottom right).

As illustrated in Fig 1, we found that psychedelic users rated their sense of connectedness as higher (*M* = 7.23, *SD* = 1.56) compared to non-users (*M* = 6.10, *SD* = 1.57; *t*(324) = -6.42, *p* < .001, *d* = 0.72). The follow up t-test for each subscale of the connectedness scale mirrored this main effect with a significant difference for connectedness to the self (*t*(324) = -4.03, *p* < .001, *d* = 0.45), the world (*t*(324) = -6.37, *p* < .001, *d* = 0.71), and others (*t*(324) = -5.52, *p* < .001, *d* = 0.62).

The exploratory finding of a positive correlation between frequency of psychedelic drug use and connectedness (*r*_*rho*_ = .248, *p* < .001) is in line with this pattern of findings. Psychedelic users who reported a higher number of psychedelic experiences also rated their connectedness as higher.

The sensitivity analyses predicting the sum score of connectedness via the variable psychedelic drug use, while controlling for gender, age, education, employment, and diagnosis indicated that the main effect psychedelic drug use was still a significant predictor (*t*(307) = 5.94, *p* < .001). Age (*t*(314) = 2.36, *p* = .019) and diagnosis (*t*(307) = -2.03, *p* = .043) were significant predictors too. Higher age was associated with more connectedness (B = 0.03) and a psychiatric diagnosis with lower connectedness (B = -0.39). This pattern of findings indicates that the reported effect of psychedelic drug use might not be observed due to sample characteristics in terms of gender, age, or participants psychiatric diagnosis.

H2a: users of psychedelic compounds show higher divergent thinking ability compared to non-users of psychedelics (H2b: the number of self-administered psychedelic compounds over the lifetime are positively associated with divergent thinking ability)

**Fig 2:**
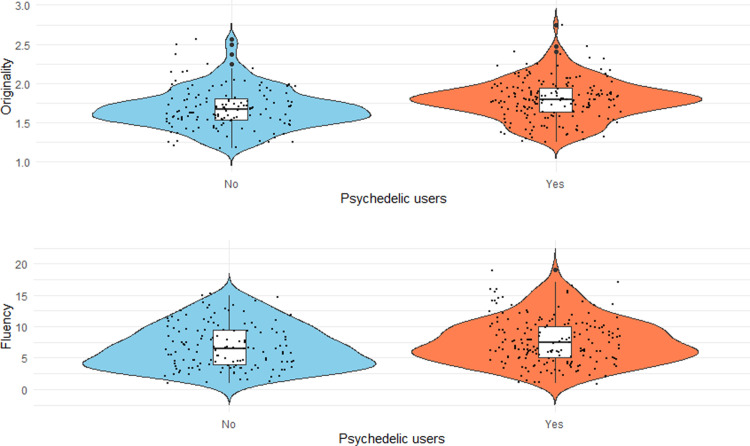
Violin plots for creative potential. We present originality as well as fluency.

As hypothesized, we found a significant difference between psychedelic users and non-users with respect to originality (*t*(324) = -3.94, *p* < .001, *d* = 0.44) and fluency (*t*(324) = -2.26, *p* = .024, *d* = 0.25; more details see Fig 2). Psychedelic users produced more original ideas (*M* = 1.79, *SD* = 0.25) and showed a higher fluency of ideas (*M* = 7.68, *SD* = 3.65) compared to non-users (originality: *M* = 1.68, *SD* = 0.25; fluency: *M* = 6.77, *SD* = 3.49).

With respect to H2b we observe a trend correlation between the number of self-administered psychedelic compounds over the lifetime (self-rated) and originality (*r*_*rho*_ = .132, *p* = .072) but not for fluency (*r*_*rho*_ = -.060, *p* = .417).

The sensitivity analyses via linear regression controlling for gender, age, employment, educations, as well as diagnosis, showed a significant effect of psychedelic drug use for originality (B = 0.10, *t*(307) = 3.31, *p = *.001) and fluency (B = 1.43, *t*(307) = 3.22, *p = *.001). We observed no significant effects of other variables on originality or fluency except lower scores of originality for older participants (B = -0.00, *t*(307) = -2.00, *p = *.046), and higher fluency scores for women compared to men (B = -0.98, *t*(307) = -2.17, *p* = .031). This indicates some robustness of findings and no strong influence of demographic sample characteristics on the main study findings.

### Exploratory analyses

#### Additional findings for real-life creativity.

The difference between psychedelic users and non-users for real-life creative activities was significant at a trend (*t*(324) = -1.84, *p* = .066, *d* = 0.21). Psychedelic users indicated more real-life creative activities (*M* = 11.69, *SD* = 5.28) compared to non-users (*M* = 10.66, *SD* = 4.44). The effect for creative achievements was not significant (*t*(301) = -1.62, *p* = .107, *d* = 0.18). The strongest effect for creative activities was observed for music (*t*(324) = -2.78, *p* = .006, *d* = 0.31) and science and engineering (*t*(324) = -3.02, *p* = .003, *d* = 0.34). Controlling gender, age, education, and diagnosis weakened the effects for music (*p* = .103) and science (*p* = .082).

Lifetime consumption (i.e., frequency) did not correlate with real-life creativity (creative activities: *r*_*rho*_ = .087, *p* = .238; creative achievements: *r*_*rho*_ = .061, *p* = .410).

#### Association of connectedness with creative ideation performance and real-life creativity.

In the exploratory analyses illustrated in Table 3, we found a significant correlation between connectedness sum score and originality but not fluency. Connectedness correlated with real-life creative activities but not with creative achievements. This pattern of findings was similar for connectedness to the self and to the world, but not for connectedness to others.

**Table 3:** Pearson correlations between connectedness scale, coincidences, and measures of creativity.

	*WCS*	*CTS*	*CTO*	*CTW*	*Synchronicity*	*Originality*	*Fluency*	*CAch*	*CAct*
*WCS*		**0.888** ***(<.001)***	**0.804** ***(<.001)***	**0.865** ***(<.001)***	**0.405** ***(<.001)***	**0.141** ***(.011)***	0.018 *(.752)*	0.005 *(.922)*	**0.162** ***(.003)***
*CTS*	**0.888** ***(<.001)***		**0.686** ***(<.001)***	**0.632** ***(<.001)***	**0.356** ***(<.001)***	**0.165** ***(.003)***	0.012 *(.832)*	-0.008 *(.892)*	**0.141** ***(.011)***
*CTO*	**0.804** ***(<.001)***	**0.686** ***(<.001)***		**0.475** ***(<.001)***	**0.238** ***(<.001)***	0.063 *(.259)*	0.012 *(.826)*	-0.011 *(.838)*	0.079 *(.154)*
*CTW*	**0.865** ***(<.001)***	**0.632** ***(<.001)***	**0.475** ***(<.001)***		**0.414** ***(<.001)***	**0.127** ***(.022)***	0.019 *(.729)*	0.025 *(.658)*	**0.177** ***(.001)***
*Synchronicity*	**0.405** ***(<.001)***	**0.356** ***(<.001)***	**0.238** ***(<.001)***	**0.414** ***(<.001)***		0.026 *(.635)*	**0.108** ***(.050)***	**0.258** ***(<.001)***	**0.366** ***(<.001)***
*Originality*	**0.141** ***(.011)***	**0.165** ***(.003)***	0.063 *(.259)*	**0.127** ***(.022)***	0.026 *(.635)*		0.033 *(.556)*	**0.170** ***(.002)***	**0.209** ***(<.001)***
*Fluency*	0.018 *(.752)*	0.012 *(.832)*	0.012 *(.826)*	0.019 *(.729)*	**0.108** ***(.050)***	0.033 *(.556)*		**0.268** ***(<.001)***	**0.253** ***(<.001)***
*CAch*	0.005 *(.922)*	-0.008 *(.892)*	-0.011 *(.838)*	0.025 *(.658)*	**0.258** ***(<.001)***	**0.170** ***(.002)***	**0.268** ***(<.001)***		**0.655** ***(<.001)***
*CAct*	**0.162** ***(.003)***	**0.141** ***(.011)***	0.079 *(.154)*	**0.177** ***(.001)***	**0.366** ***(<.001)***	**0.209** ***(<.001)***	**0.253** ***(<.001)***	**0.655** ***(<.001)***	
*Computed correlation used pearson-method with listwise-deletion.*									

Furthermore, connectedness (sum score as well as sub scores) was positively related with synchronicity experiences. Synchronicity experiences correlated with creative activities, creative achievements, and fluency, but not with the originality of ideas. The difference between psychedelic drug users and non-users with respect to synchronicity experience was significant at a trend (*t*(324) = -1.81, *p* = .071, *d* = 0.20). We observed a small effect with higher synchronicity in the group of users (*M* = 4.01, *SD* = 0.81) compared to non-users (*M* = 3.84, *SD* = 0.81). When controlling gender, age, education, and diagnosis, this effect remained unaffected.

The positive relationships of originality and fluency with creative activities and achievements indicate the validity of creativity assessments (see Table 3).

#### Connectedness as a mediator of the effect of psychedelic use on creativity (i.e., originality and fluency).

Connectedness (sum score) did not mediate the link between psychedelic drug use and originality (B = 0.01, CI95% = [-0.01-0.03], *p* = .250). However, for connectedness to the self the indirect effect (B = 0.01, CI95% = [0.00-0.03], *p* = .028) and the direct effect (B = 0.10, CI95% = [0.04-0.15], *p* < .001) were significant. Approximately 13% of the total effect (B = 0.11, CI95% = [0.05-0.16], *p* < .001) was mediated through connectedness to the self. For connectedness to the world and connectedness to others we observed no indirect effect (CTW: B = 0.01, CI95% = [-0.01-0.03], *p* = .336; CTO: B = 0.00, CI95% = [-0.00-0.02], *p* = .962). For fluency we did not find a mediation effect for any connectedness score (e.g., sum score; indirect effect: B = -0.07, CI95% = [-0.34-0.20], *p* = .648).

#### Psychedelic use, connectedness, creativity, well-being, and life satisfaction.

The final exploratory analyses showed that psychedelic users did not show higher life satisfaction (*t*(307) = 1.67, *p* = .096) and well-being (PA: (*t*(307) = -0.30, *p* = .767); NA: (*t*(307) = -0.99, *p* = .323). When controlling gender, age, education, and diagnosis, all effects remained non-significant. However, as illustrated in Table 4, life satisfaction correlated positively with connectedness, synchronicity experiences, and parameters of creativity (i.e., fluency) as well as with positive (and negative) affect. Interestingly real-life creativity correlated positively with PA (and originality and fluency at a trend).

**Table 4:** Pearson correlations of connectedness and creativity with life satisfaction and wellbeing (PA, NA).

	*WCS*	*CTS*	*CTO*	*CTW*	*Synchronicity*	*Originality*	*Fluency*	*CAch*	*CAct*	*PA*	*NA*	*SWLS*
*PA*	**0.362** ***(<.001)***	**0.312** ***(<.001)***	**0.266** ***(<.001)***	**0.339** ***(<.001)***	**0.233** ***(<.001)***	0.094 *(.099)*	0.105 *(.064)*	**0.197** ***(<.001)***	**0.281** ***(<.001)***		**-0.295** ***(<.001)***	**0.504** ***(<.001)***
*NA*	**-0.312** ***(<.001)***	**-0.220** ***(<.001)***	**-0.392** ***(<.001)***	**-0.214** ***(<.001)***	-0.039 *(.491)*	0.094 *(.099)*	0.093 *(.103)*	**0.187** ***(.001)***	**0.124** ***(.030)***	**-0.295** ***(<.001)***		**-0.490** ***(<.001)***
*SWLS*	**0.301** ***(<.001)***	**0.281** ***(<.001)***	**0.339** ***(<.001)***	**0.184** ***(.001)***	**0.209** ***(<.001)***	0.049 *(.391)*	**0.163** ***(.004)***	0.004 *(.951)*	0.110 *(.054)*	**0.504** ***(<.001)***	**-0.490** ***(<.001)***	

Computed correlation used pearson-method with pairwise-deletion.

H3: a stronger sense of connectedness is associated with diminished addictive behavior

As preregistered there was a significant difference between addictive behavior with respect to connectedness (*t*(301) = 2.74, *p* = .007, *d* = 0.32). Addicted participants (n = 121) showed lower scores of connectedness *M* = 6.47 (*SD* = 1.70) compared to non-addicted participants (n = 182; *M* = 6.99, *SD* = 1.58). Furthermore, self-attributed addicted people showed lower life-satisfaction (*t*(301) = 3.75, *p* < .001), PA (*t*(301) = 2.21, *p* = .028), and higher NA (*t*(301) = -6.01, *p* < .001).

## Discussion

As hypothesized in the preregistration [48], this study showed that people who reported self-determined psychedelic drug experiences (within their lifetime) achieved higher creative potentials in terms of originality and fluency (see, e.g., [5,13,15,58]; but see, e.g., [12] for long term effects) and at the same time psychedelic drug users rated their sense of connectedness as higher (in all three subscales; [19]). Furthermore, the more often people have used psychedelic drugs the more original (but not fluent) were their ideas (at a trend) and the higher was the sense of connectedness (in all three subscales). The additionally conducted exploratory analyses indicated that the sense of connectedness correlated positively with people’s creative potential (in terms of originality but not fluency). The consequently calculated mediation analysis suggests that the link between psychedelic drug use (users vs non-users) and originality was partly due to the sense of connectedness to the self (but not to the world and to others).

These study findings are in line with previous online surveys indicating higher creative ideation performance as well as self-reported benefits for creativity associated with psychedelic drug use [13,15]. Prochazkova [14] found acute and positive effects of microdosing on divergent thinking in natural open labeled microdosing events. The current study can add the finding that connectedness, especially to the self and to the world, could potentially represent a psychosociological factor in the long-term influence of psychedelic drug use on creative cognition. Additionally, we can conclude from the present pattern of findings that associations with real-life creative behavior might be weaker compared to creative ideation performance. However, if we take the trend effect seriously, we might speculate that psychedelic drug use not only impacts creative ideation skills but also everyday life creative activities. People who use psychedelic drugs seem to more often engage in creative activities such as playing music or spending time with open scientific and engineering problems. This is a novel finding, indicating that besides better creative cognition, psychedelics might have long-term effects on creative activities in real-life settings. Nevertheless, controlling for group characteristics weakened these effects and we did not find convincing evidence for a strong difference in creative achievements between psychedelic drug users and non-users. However, one reason for these weaker effects might be that we did not investigate people with elevated levels of creative achievements in the present study.

Similar to previous work [39], we found a link between synchronicity experiences and real-life creativity (creative activities and achievements) and the absence of a relationship with creative ideation performance in terms of originality [47]. But we found a positive association with the fluency component of creative potential. This pattern of findings is in accordance with Kéri [23], who reported a relationship between creative achievement and unusual experiences [59]. Furthermore, in line with literature we observed a positive link between synchronicity experience and positive affect and life satisfaction [44]. The experience of meaningful coincidences was slightly higher in psychedelic users (at a trend), which was not affected by the sample characteristics. Additionally, the association between synchronicity experiences and the sense of connectedness is in line with Watts [19], who showed a link between connectedness and mystical experiences.

Interestingly, we found an association between connectedness (specifically to the self and the world) and indicators of creativity, such as originality and engagement in creative activities. This integrates in existing literature indicating associations of creativity with connectedness to nature [33], as well as self-regulation and emotional skills [35,36,60]. However, the absence of a significant association for connectedness to others contrasts with research indicating that an individual’s social network [22,23], feelings of loneliness [25], and social support [26] are linked to creativity. Notably, the elevated level of connectedness to others within our sample displayed minimal variation, which might decrease the potential to detect meaningful relationships. Additionally, social exclusion, indicated by lower connectedness to others, can also increases facets of creativity such as malevolent creativity [61]. It seems plausible that this might diminish the expected positive relationship between connectedness to others and creativity. Taken together, our findings suggest that perspective taking might serve as a common factor between creativity and connectedness [33,34], which also goes along with connectedness to nature [62].

In accordance with this, the observation that connectedness to the self partly mediated the association between psychedelic drug use and originality strengthens the idea that connectedness serves as an important psychological factor. The partial mediation is in accordance with Watts [19], who showed that psilocybin induced changes in connectedness can explain changes in depressive symptoms. Psychedelics might increase mood and connectedness which in turn changes people’s creative potential [17,18]. However, contrary to this assumption we did not find convincing evidence for a link between positive (and negative) affect and psychedelic drug intake, although PA was related with all facets of creativity such as creative activities and achievements (and originality and fluency at a trend). This is in accordance with literature [63,64], frequently reporting a close link between positive affect and originality of ideas as well as creative activities [39,53,65–68].

Positive affect and life satisfaction were associated with connectedness and synchronicity experiences. Furthermore, life satisfaction showed a relationship with the fluency of ideas but failed to show a positive relationship with creative activities, which was only marginally significant. Furthermore, psychedelic drug users did not show higher life satisfaction. Taken together, perceiving meaning, and a connection to the self, the world and to others seem important for life satisfaction [19,44,69], but not the use of psychedelic drugs per se. Contrary to previous work, the present cross-sectional online survey did not confirm the positive link between psychedelic drug use and variables of well-being and life satisfaction [2,18]. This pattern of findings might have taken place because of the cross-sectional study design, which does not capture the acute and more short-term effects of substance use.

As preregistered [48], we found that people who reported potential addictions (i.e., behavior or substance use; [55]) showed lower scores of connectedness. Lower positive affect, higher negative affect, and reduced life satisfaction in the group classified as potentially addicted (due to self-report on a screening instrument) accompanied this pattern of findings, which is even more interesting when taking the higher connectedness in psychedelic drug users into account. This strengthens the view that connectedness is a crucial factor for a better mood and higher life satisfaction (reduced in people who perceive themselves as more addicted).

## Limitation

One limitation of this study is the cross-sectional design, which precludes the establishment of a causal relationship between variables. As a result, alternative causal directions are plausible, such as the possibility that a sense of connectedness may heighten the propensity for psychedelic drug consumption. Additionally, individuals with higher creative abilities may have an increased inclination to use psychedelics to seek existential meaning or to augment their creative abilities further [70]. Nevertheless, available evidence suggests the enhancing effects of psychedelics on creative ideation performance ([7,13]; for reviews see, e.g., [1,17]) as well as the feelings of connectedness [19,71]. The cross-sectional findings of this study are therefore well in line with literature suggesting increases in creativity and sense of connectedness (for critical reviews on psychedelic research see, e.g., [12,16]).

Since we did not experimentally induce substance intake but investigated the cross-sectional relationships between self-determined psychedelic drug experiences, connectedness, creativity, and life satisfaction, self-selection biases (only interested people participated in this study) might have led to an overestimation of effects [17]. However, we measured creative ideation performance objectively, by means of a performance task. Furthermore, the frequency of psychedelic drug use correlated with originality (at a trend), which further increases the trustworthiness of findings. In contrast to experimental approaches, the cross-sectional design of this study adds observations in more naturalistic settings to literature (see also [13,40]). The present cross-sectional study targeted to investigate potential links between self-determined psychedelic experiences and creative ideation as well as real-life creative behavior such as creative activities and achievements, which constitutes an additional novel aspect.

A further limitation is that we did not assess openness and curiosity in this study. Since both personality traits are linked with creativity [72,73] and psychedelic drug use [2,13] these traits might serve as alternative explanations why psychedelic users are more creative than non-users [46]. Future studies should additionally assess and explore openness and curiosity in more detail as further potentially mediating variables beside the psychological factors of mood, pattern break, and feelings of connectedness.

Additionally, both groups (psychedelic users and non-users) not only differed with respect to the variables of interest but also showed substantial differences with respect to age, gender, education, and psychiatric diagnosis. We statistically controlled for these parameters in more complex and additional sensitivity analyses. Importantly, the main results remained significant and stable for these analyses. Therefore, the reported results should be characteristics of psychedelic users vs. non-users in general and are not due to the specific group characteristics of our sample. Nevertheless, although we preregistered the main hypotheses, the study findings need replication.

## Conclusions

This cross-sectional online survey showed that people who use psychedelic drugs feel more connected (to the self, others, and the world). They produced more creative ideas (in terms of originality and fluency), and they showed a trend for more creative activities (but not creative achievements). Consequently, psychedelic drug users not only have a higher creative potential, but they also behave differently in their daily lives. They seem to play music more often and are more frequently engaged in working on open-ended scientific and engineering problems [46]. However, psychedelic drug users did not differ from non-users in terms of life satisfaction or well-being [18]. While psychedelic drugs carry the potential for maladaptive effects, such as increased acute fear, heightened anxiety during use, and the risk of psychotic episodes [70,74], these effects may partly arise from the heightened creativity that allows the mind to imagine threats and dangers from novel perspectives. On the other side of the same coin, the present study demonstrates that psychedelic drug users showed positive outcomes such as higher creative potential, more creative activities, and stronger feelings of connectedness.
